# Serving Vietnam Veterans Hospitalized Outside the VA System: A Scoping Review of Presumptive Service-Related Illnesses and Presentations

**DOI:** 10.1007/s11606-025-09601-8

**Published:** 2025-05-15

**Authors:** Robert Miller, Attila Nemeth, J. Luke Taggart, Mary Ann Kirkconnell Hall, Joyce Akwe

**Affiliations:** 1 New Orleans VA Medical Center, New Orleans, LA USA; 2 Tulane University Medical School, New Orleans, LA USA; 3 VA Northeast Ohio Healthcare System, Cleveland, OH USA; 4 Case Western Reserve University School of Medicine, Cleveland, OH USA; 5 Division of Hospital Medicine, Department of Medicine, Emory University School of Medicine, Atlanta, GA USA; 6 Atlanta VA Health Care System/VISN 7 Clinical Resource Hub, Atlanta, GA USA

**Keywords:** scoping review, Vietnam Veterans, hospital medicine, service-related conditions

## Abstract

**Background:**

Recent legislation, the MISSION Act and the PACT Act, expanded access to and utilization of non-Department of Veterans Affairs (VA) health care; more Veterans now receive care from non-VA healthcare providers. Hospitalists outside the VA may be less familiar with Veterans’ service-related exposures and presumptive service-related conditions. We aimed to summarize research findings on service-related exposures and conditions among Vietnam War Veterans.

**Methods:**

Using Arksey and O’Malley’s methodological framework for scoping reviews, we searched PubMed, EMBASE, and Web of Science databases in June 2023. References were imported into EndNote and screened using Covidence collaborative review software. Two reviewers assessed eligibility, with disagreements resolved by a third, then one extracted data. We included papers published in 1998 or later focused on US Vietnam Veterans, excluding genetic/modeling studies, study protocols, case reports/series, clinical trials, and papers without relevance to hospital medicine.

**Results:**

We identified 1185 papers; 251 were duplicates, 450 were excluded through title/abstract review, and 335 were excluded after full-text review. A total of 149 studies were included. The exposures mentioned most frequently were Agent Orange/unspecified herbicides (*n* = 55), violence/combat (*n* = 14), and infectious disease (*n* = 9). The most common conditions were PTSD (*n* = 39), neuropsychiatric conditions (*n* = 35), cancer (*n* = 19), metabolic/endocrine disease (*n* = 11), and neurological dysfunction (*n* = 11). Overall mortality was addressed in 13 studies.

**Conclusions:**

The current literature highlights numerous service-related exposures and conditions recognized by the VA, which may assist hospitalists caring for Vietnam Veterans outside the VA.

**Supplementary Information:**

The online version contains supplementary material available at 10.1007/s11606-025-09601-8.

## INTRODUCTION

Recent legislation, particularly the MISSION Act and the PACT Act, has expanded access to healthcare services for Veterans, enabling them to receive care from non-Veterans Affairs (VA) providers. This has increased the number of Veterans seeking care outside the VA system. However, hospitalists and healthcare providers in non-VA settings may have limited familiarity with Veterans’ unique service-related exposures and presumptive conditions, particularly those who served during the Vietnam War. Service-related health issues often require specialized knowledge to ensure appropriate care. While presumptive conditions require referral to a VA facility, this scoping review aims to assist non-VA hospitalists to recognize conditions linked to toxic exposures and take appropriate steps to ensure optimal patient care.

To understand service-related conditions specific to US Veterans of the Vietnam War, their unique conditions and circumstances should be noted. Vietnam’s wet, tropical climate and hazardous geography—characterized by dense jungles, mountains, and rivers—complicated travel, health, and equipment maintenance practices. Utilization of defoliants and chemicals such as napalm were also significant hazards. Troops faced unfamiliar guerrilla tactics including ambushes and booby traps. Socially, the controversial nature of the war and Veterans’ negative reception by some of the American public on return home further shaped their experiences. These environmental, tactical, and social factors uniquely impacted the health of US troops during and after their Vietnam War service.

## OBJECTIVES

We sought to identify and summarize reported toxic exposures and service-related^1^ conditions among Vietnam War Veterans, as documented in the literature. By familiarizing non-VA hospitalists with these key presumptive conditions, we aim to enhance their ability to recognize and diagnose these issues in community healthcare settings. Furthermore, this review provides non-VA hospitalists practical insights for managing and treating Veterans with service-related conditions and when to refer to VA facilities. Ultimately, our goal is to bridge knowledge gaps between VA and non-VA hospitalists, improving overall quality of care for Vietnam War Veterans in the community.

## METHODS

We used Arksey and O’Malley’s methodological framework for scoping reviews (Supplemental Appendix 1 for a completed Preferred Reporting Items for Systematic reviews and Meta-Analyses extension for Scoping Reviews (PRISMA-ScR) Checklist); the protocol is registered in Open Science Framework at https://osf.io/c4nm3/.^2,3^ We searched PubMed, Google Scholar, EMBASE, and Web of Science from January 1998 to June 2023, applying inclusion and exclusion criteria (see Table 1 for inclusion and exclusion criteria and Supplemental Appendix 2 for a sample search strategy). Eligible studies were published in 1998 or later in English and included US Veterans of the Vietnam War. Additional inclusion and exclusion criteria were developed following discussion and consensus to reflect assessment of utility based on our clinical experience as VA hospitalists.

**Table 1:** Inclusion and Exclusion Criteria

Inclusion criteria	Exclusion criteria
Relevance to the inpatient setting* Published in 1998 or later Includes US Veterans of the Vietnam War Published in English	Solely outpatient setting Single case reports or small case series Editorials/Letters to the Editor Published before 1998 Non-US Veterans No physical presence in Vietnam Non-human subjects (service dogs, murine models) History/political science Focus on spouses, not Veterans Studies of patient experiences within the VA system or patient utilization of VA services Development of psychometric instruments Clinical trials of medications, devices, or psychological treatments Genetic association studies Statistical models of association with no prevalence/exposure data

^*^Relevance to the inpatient setting was a subjective criterion determined by the 4 physician reviewers based on their years of experience as hospitalists in the VA setting

Results were imported into EndNote, and duplicates were removed before transferring the dataset to Covidence (Veritas Health Innovation Ltd, Melbourne, Australia). A team of four VA hospitalists, supported by a medical writer, conducted a rapid review of titles, abstracts, and full texts for eligibility. Data were extracted using a standardized form (Supplemental Appendix 3). Following scoping review methodology, we did not assess study quality or evidence strength.

## RESULTS

**Figure 1:**
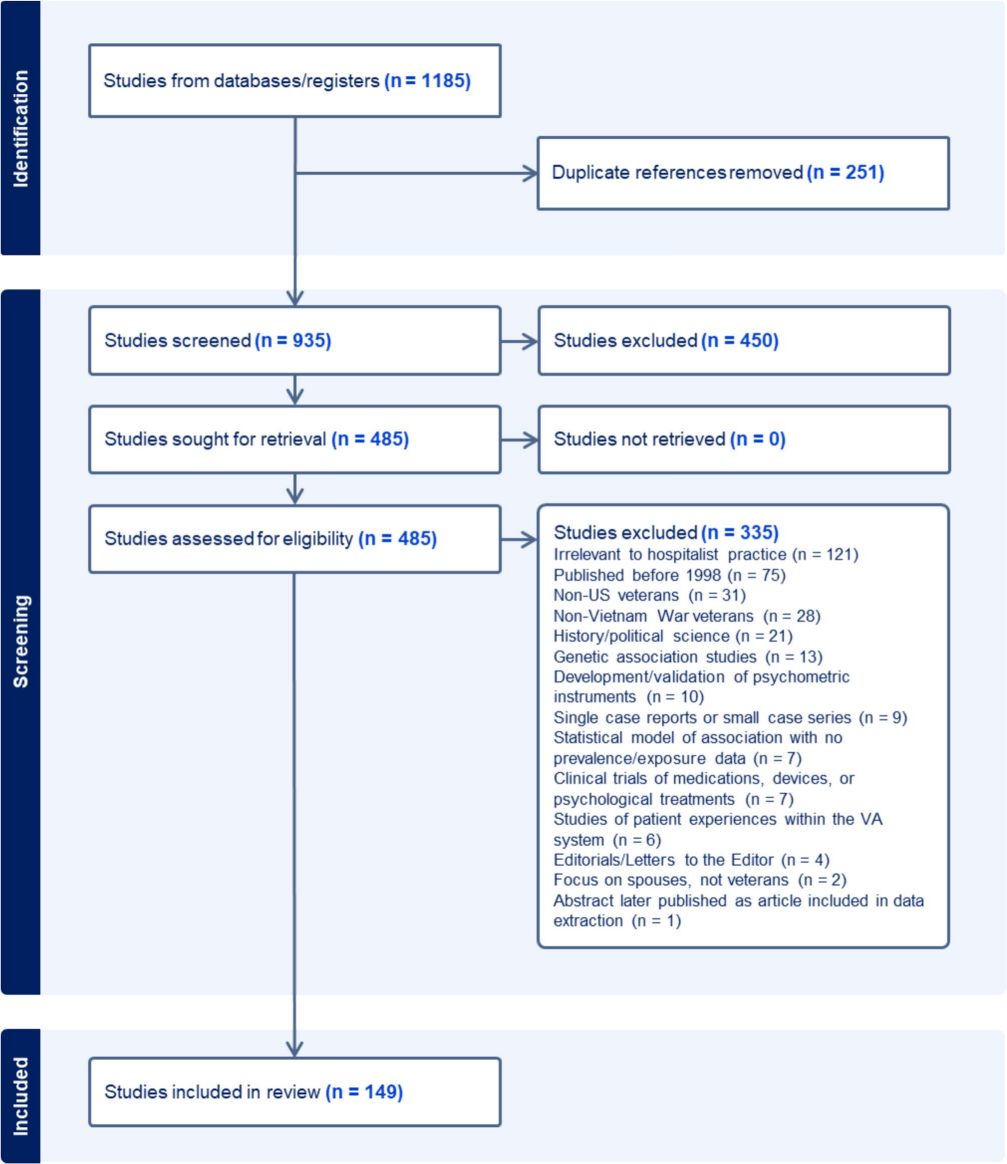
PRISMA diagram: PRISMA flow diagram illustrating the study selection process. Out of 1185 studies initially identified from database searches, 251 duplicate references were removed, leaving 935 studies screened by title and abstract. After screening, 450 studies were excluded, and 485 studies were assessed for eligibility. Of these, 335 studies were excluded due to various criteria, including irrelevance to hospitalist practice, publication date prior to 1998, non-US Veteran populations, and focus on non-Vietnam War Veterans. The final review included 149 studies relevant to service-related exposures and conditions among Vietnam War Veterans.

Overall, 1185 articles were imported into Covidence for screening; 251 duplicates were removed (see Fig. 1). During title and abstract review, 450 articles were excluded; we iteratively expanded exclusion criteria, e.g., excluding genetic association studies and studies using Veteran cohorts to assess conditions not connected to service exposures. Among the 485 studies selected for full-text review, 335 were excluded. Overall, 149 articles were included in the scoping review (Supplemental Appendix 4). Data extracted included population/setting; comparison group (if any); study type; study results/conclusions; exposures; medical conditions; and other pertinent information (Supplemental Appendix 3).

**Figure 2:**
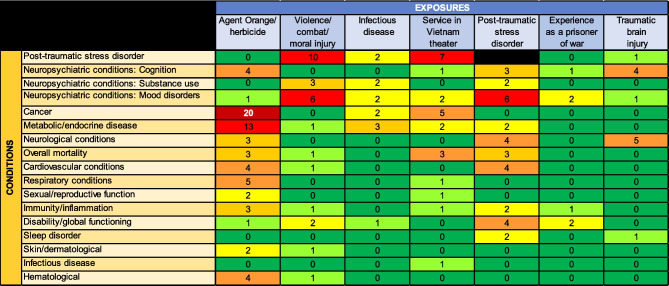
Heat map of articles describing potential associations between exposures (horizontal axis) and conditions (vertical axis): Heatmap depicting the frequency of reported service-related exposures and associated health conditions among Vietnam War Veterans. Rows represent health conditions (e.g., PTSD, neuropsychiatric disorders, cancer), while columns indicate specific exposures (e.g., Agent Orange, combat experience, infectious disease). Color intensity reflects the frequency of mention in the literature, with red indicating the highest frequency and green the lowest. Note: Per methodological guidelines for scoping reviews, we did not assess the strength of evidence for each study included, but instead noted their relative frequency to demonstrate where research to date has been concentrated.

We found that the exposure most frequently described in the literature was Agent Orange, and the condition described most often was post-traumatic stress disorder, followed by neuropsychiatric conditions (see Fig. 2 for a heat map depicting the relative frequency of articles examining exposures and conditions). Below, we present a synthesis of the literature in narrative and table format, divided into domains of exposures and conditions in related systems. A high-level summary table of considerations and implications for internists is included as Table 7 in the “Results” section.

### Documented Conflict-Related Risks and Exposures Experienced by US Veterans of the Vietnam War

#### Herbicides/Agent Orange

Agent Orange is one of several phenoxy herbicides and defoliant chemicals used extensively in Vietnam and nearby areas to clear vegetation. Applied primarily by aircraft and other vehicles, nearly 20 million gallons of herbicides were sprayed during 1962–1971. Agent Orange was commonly contaminated by a manufacturing byproduct—2,3,7,8-tetrachlorodibenzo-p-dioxin, known also as TCDD or simply “dioxin”—later established as a toxin and potent carcinogen. The health effects of Agent Orange have been extensively investigated.

We identified a significant body of research—45 articles—examining impacts of Agent Orange exposure among US Vietnam Veterans with health outcomes carrying implications for inpatient hospital medicine practices; the most salient are summarized in Table 2.

**Table 2:** Articles Examining Impacts of Agent Orange Exposure

Author(s)/year	Condition/variable	Article summary
Ansbaugh et al. 2013^4^	Prostate cancer	• Single-center historical cohort analysis • 2720 Veterans who underwent prostate biopsy • Agent Orange exposure was associated with a 52% increase in overall risk of prostate cancer detection (adjusted odds ratio (aOR) 1.5), a 75% increase in risk of high-grade prostate cancer, and a 2.1-fold increase in Gleason 8 or high cancer at diagnosis • No significant association with increase in low-grade prostate cancers
Barnes et al. 2019^5^	Dementia	• Retrospective cohort study • Random sample of 498,749 Vietnam-era Veterans • After adjusting for demographics and comorbidities, Veterans with Agent Orange exposure were 20% more likely to have a dementia diagnosis (adjusted hazard ratio 1.2)
Barrett et al. 2001^6^	Cognitive decline	• Prospective study • 937 Operation Ranch Hand Veterans (Air Force members who sprayed Agent Orange herbicide during Vietnam War) with elevated blood dioxin levels • Veterans with highest Agent Orange exposure had highest risk of cognitive function decline, especially in memory functions
Boyle et al. 2006^7^	Various health outcomes including coronary heart disease (CHD)	• Prospective, case–control study • 2105 Operation Ranch Hand Veterans • Adjusting for CHD risk factors, depression, anxiety, hostility, and trait anger were significant predictors of incident CHD
Burton et al. 1998^8^	Acne, chloracne	• Outpatient, longitudinal, prospective study • 952 in the study group of participants in Operation Ranch Hand and 1281 in a control group of Air Force Veterans not involved in this operation • No meaningful association between dioxin exposure or subsequent serum levels and prevalence or development of acne or chloracne
Chamie et al. 2008^9^	Prostate cancer	• Retrospective cohort study • 13,144 men, including 6214 exposed to Agent Orange • Exposed men had twice the odds of prostate cancer (OR 2.2) compared to unexposed men. They were diagnosed at a younger age, had a twofold increase in Gleason scores of 8–10, and were more likely to present with metastatic disease (13% vs. 4% in unexposed men)
Cypel et al. 2018^10^	Chronic obstructive pulmonary disease (COPD)	• Retrospective cohort study • 3193 US Army Chemical Corps Veterans • While spirometric parameters did not differ by Agent Orange exposure, self-reported COPD was significantly associated with Agent Orange exposure (adjusted odds ratio (aOR) = 1.82)
Cypel and Kang 2010^11^	All-cause mortality, respiratory system disease, respiratory system cancer	• Retrospective cohort study • 2872 Army Chemical Corps Vietnam Veterans compared to a matched, non-Vietnam cohort to assess disease-related mortality with subset analysis done on those who reported spraying herbicides versus those who did not • Statistically significant excess mortality was observed in Army Chemical Corps Veterans for COPD (ARR 4.8)
de la Monte and Goel 2022^12^	Peripheral neuropathy	• Review • Higher levels of Agent Orange exposure increased the risk of developing peripheral neuropathy as both a primary condition or as a complication of diabetes. It could also contribute to the pathogenesis of neurodegenerative diseases such as dementia
Frumkin 2003^13^	Various cancers	• Review • There is sufficient evidence of an association between Agent Orange exposure and soft tissue sarcoma, non-Hodgkin lymphoma, Hodgkin disease, and chronic lymphocytic lymphoma. Other discussed cancers have limited or insufficient evidence of an association
Gupta et al. 2006^14^	Benign prostatic hyperplasia	• Longitudinal prospective cohort case–control study • 971 U.S. Air Force Veterans involved in Operation Ranch Hand compared to 1266 Air Force Veterans who did not spray herbicides • Higher serum TCDD (Agent Orange ingredient) levels are associated with decreased risk of BPH
Kang et al. 2006^15^	Diabetes, heart disease, hypertension, and chronic respiratory diseases	• Cohort study • 1499 Army Chemical Corps Veterans with Agent Orange exposure compared to 1428 non-Vietnam Veterans assigned to chemical operations jobs who were surveyed on their health diagnoses which were analyzed in association with serum levels of dioxin • Significantly elevated odds ratios for diabetes (1.5), heart disease (1.5), hypertension (1.3) and chronic respiratory diseases (1.6) in those who sprayed herbicides
Ketchum and Michalek 2005^16^	All-cause and circulatory disease mortality	• Retrospective cohort study • 1262 Operation Ranch Hand Veterans with over 19,000 other Veterans and assessing relative risk of all-cause death • Relative risk for all-cause death was borderline significantly increased (RR 1.15) and risk of death due to circulatory system diseases was significantly increased among enlisted ground crew workers Operation Ranch Hand who had the highest exposure levels compared to pilots, flight engineers, and others (RR 1.7)
Landgren et al. 2015^17^	Monoclonal gammopathy of undetermined significance (MGUS)	• Prospective cohort study tested for MGUS • Serum specimens of the Air Force Health Study and compared this to relevant exposure data—it included 479 Ranch Hand Veterans with comparison group of 479 other Veterans • Prevalence of MGUS was higher (7.1%) in Ranch Hand Veterans compared to comparison Veterans (3.1%) for an odds ratio of 2.4. Higher dioxin levels were found in 47% of the Ranch Hand Veterans compared to only 2.5% of comparison Veterans
Michalek et al. 1999^18^	Serum dioxin, insulin, fasting glucose, sex hormone-binding globulin	• Cohort study • 871 Operation Ranch Hand Veterans who sprayed/handled Agent Orange and 1121 comparison Veterans who served in the Vietnam theater but did not spray herbicide, categorizing both groups into low, medium, and high exposure strata • In non-diabetic Vietnam Veterans with high dioxin exposure, insulin levels were more elevated and a shift in the sex hormone-binding globulin-insulin relationship could indicate the body’s attempt in compensating metabolically. These findings suggest Agent Orange exposure may disrupt normal insulin regulation and heighten long-term risk for type 2 diabetes
Michalek et al. 2001^19^	Peripheral neuropathy	• Outpatient, longitudinal cohort study • 761 Operation Ranch Hand Veterans who were stratified into exposure categories based on serum dioxin levels and underwent serial peripheral nerve function assessments over fifteen years • Those in the high exposure group had a statistically significant increased risk of diagnosed peripheral neuropathy. Pre-clinical diabetes is noted as a possible confounder
Michalek et al. 2003^20^	Psychological problems	• Cohort study • 1109 Operation Ranch Hand Veterans and 1493 matched, non-Ranch Hand comparison Veterans • Few consistent psychological abnormalities associated with serum dioxin levels
Mossanen et al. 2017^21^	Bladder cancer	• Review article • Air Force Health Study participants • Studies exist that suggest an association between dioxin and occurrence of bladder cancer and increased mortality from bladder cancer
National Academies of Science, Engineering, and Medicine 2017^22^	Varied	• Epidemiologic study review • Updates the list of Vietnam-era herbicide associated medical conditions per the National Academies of Sciences, Engineering, and Medicine. It is acknowledged that US Military Veterans in Vietnam 1962–1971 who could have been exposed to herbicides could be at risk of potential associations of several health outcomes including soft tissue sarcoma, non-Hodgkin lymphoma
Nwanaji-Enwerem et al. 2020^23^	Serum dioxin levels and sperm DNA methylation age	• Cross-sectional study • 37 male Air Force Health Study participants exposed to Agent Orange using a novel DNA methylation-based measure of sperm age to examine the relationship with serum dioxin levels • Dioxin exposure is associated with increases in sperm methylation age, suggesting implications of Agent Orange exposure and male fertility
Pavuk et al. 2006^24^	Prostate cancer	• Case–control study • 2516 Veterans (1019 Operation Ranch Hand Veterans and 1497 non-Ranch Hand comparison Veterans) • There was a statistically significant increase in mean TSH levels in those with high dioxin exposure, but no significant increase in thyroid disease was shown, suggesting at least an effect of Agent Orange on thyroid hormone metabolism
Shah et al. 2009^25^	Clinicopathological characteristics and outcomes after radical prostatectomy (RP) in patients with prostate cancer	• Cohort study • 1495 Veterans with prostate cancer and previous exposure to Agent Orange • Patients with Agent Orange exposure who underwent radical prostatectomy were at increased risk of biochemical progression and had shorter PSA doubling time
Williams et al. 2023^26^	Bladder cancer	• Retrospective cohort study • 629,907 Veterans with Agent Orange exposure compared to 1,888,019 matched Veterans without such exposure • Modest but statistically significant increased risk of bladder cancer in male Vietnam Veterans exposed to Agent Orange (HR 1.04) but no association with increased aggressiveness of bladder cancer
Young et al. 2004^27^	Various	• Literature review on dislodgeable foliar residue • Only 8% of the herbicide remained on plant leaves one hour after application, dropping to 1% after 24 h. Human volunteer studies revealed that after 2 h of direct skin contact, only 0.15–0.46% of 2,4,5-T, a component of Agent Orange, was absorbed and excreted in urine
Young et al. 2004^28^	Various	• Historical and procedures review • Herbicide spray missions during the Vietnam War, such as Operation RANCH HAND which accounted for 95% of defoliant use, were meticulously planned to avoid areas with friendly forces, with missions being canceled or not approved if such forces were present. Consequently, very few friendly troops were exposed to spraying from fixed-wing aircraft, helicopters, or surface operations, thanks to strict policies and procedures guiding the approval and execution of these missions
Zafar and Terris 2001^29^	Prostate Cancer	• Longitudinal case–control study • 400 Veterans to assess the rate of prostate cancer in Veterans who reported a history of Agent Orange exposure compared to Veterans who denied such exposure • There was no statistically significant difference in PSA, prostate cancer, or cancer grade in patients with Agent Orange exposure versus controls

Veterans’ exposure to Agent Orange varied widely. Many studies suggest those directly involved in spraying as participants in the Air Force Operation Ranch Hand earlier in war and for longer durations were at greatest risk of developing associated conditions.^27^ While herbicide spray missions were carefully planned to avoid direct contact with friendly forces,^28^ many self-reported exposures occurred. Indeed, while all Veterans should be asked about their exposure histories, VA policy has established Veterans are presumed exposed if they had “boots on the ground.”

Hospitalists should be vigilant for symptoms concerning for neoplastic processes in patients with known Agent Orange exposure as early detection and intervention can affect clinical outcomes. Seventeen articles focused on carcinogenic effects of Agent Orange. Although studies support an increased incidence of soft tissue sarcoma, non-Hodgkin lymphoma, Hodgkin lymphoma, and chronic lymphocytic leukemia with phenoxy herbicides, increased incidence in Vietnam Veterans exposed to Agent Orange is less established^9,13^ due to low numbers of highly exposed subjects.

Two studies found prostate cancer risk to increase with Agent Orange exposure^30^ with earlier development, more aggressive variants, and higher-grade prostate cancer at diagnosis,^4,9^ but a longitudinal case–control study of 400 Veterans found no significant relationship between prostate cancer and Agent Orange exposure.^29^A cohort study of 1495 Veterans with prostate cancer and Agent Orange exposure found a predisposition to adverse outcomes post-radical prostatectomy, regardless of race.^25^

Studies also support increased bladder cancer incidence^26^ and mortality from bladder cancer.^21^ Limited evidence suggests an association with monoclonal gammopathy of undetermined significance (MGUS)^17^ and melanoma.^30^

Other papers investigated non-cancerous conditions related to Agent Orange exposure, noting increased risk of peripheral neuropathy with high levels of exposure.^12,19^ Veterans exposed to Agent Orange were > 20% more likely to be diagnosed with dementia,^5^ potentially at earlier ages.^31^ Veterans who sprayed herbicides had increased risk of hypertension and heart disease,^15^ and some Ranch Hand Veterans had increased relative risk of death caused by circulatory system diseases.^16^ Among dermatological conditions, though evidence of potential association with Agent Orange chloracne is inconclusive,^8^ it is recognized as a presumptive service-related condition.^1^

No conclusive link has been found between Agent Orange exposure and non-cancerous respiratory diseases; a potential association between herbicide exposure and self-reported COPD was not supported by spirometry data.^10^ Another cohort study evaluated spirometry restrictions in 468 Vietnam War Veterans exposed to herbicides during chemical operations confirmed via serum levels of 2,3,7,8-tetrachlorodibenzo-p-dioxin, but found no higher prevalence of restrictive lung disease in Veterans involved in chemical operations.^32^

#### Violence/Combat Exposures

Thirteen studies addressed impacts of exposure to violence and/or combat; eight^33–40^ focused on PTSD (Tables 4 and 5 below); four^41–44^ focused broadly on general cognition and mental health (Table 6); and one^45^ focused on TBI.

Most articles examining outcomes associated with service in the Vietnam theater (versus service during the Vietnam War area outside Vietnam) found that in-theater service was associated with more mental health problems (notably PTSD^46–48^ and nightmares^49^), increased rates of cancer (likely due to herbicide exposures),^50^ and higher overall mortality.^50,51^

#### Infections

Ten studies on infections in Vietnam War Veterans were identified; the three most salient examined hepatitis C virus (HCV; Table 3). One study of 2638 HCV-infected Vietnam era Veterans compared to non-Veterans identified men having sex with men as a significant risk factor among Veterans. However, Veterans were more inclined to attribute other exposures as the infection source.^52^ Another study of 22,341 HCV-infected Vietnam Veterans found a higher prevalence of major psychiatric disorders compared to controls.^53^ Demographic and clinical variations were also noted: African Americans with HCV exhibited higher rates of obesity, alcohol abuse, diabetes, and specific HCV genotypes compared to non-Hispanic Whites, while Hispanics had higher proportions of diabetes, alcohol abuse, obesity, and HIV co-infection.^54^

**Table 3:** Articles Examining Impacts of Infectious Disease Exposure

Author(s)/year	Exposure	Condition	Article summary
Boscarino et al. 2014^52^	Vietnam War service	Hepatitis C	• Case–control study • 2638 hepatitis C-positive Vietnam era Veterans vs. non-Veterans to investigate the reasons why hepatitis C was more prevalent in this population • Comparison of specific risk factor differences for HCV infection by Veteran status suggested that no HCV risk factor was associated with Vietnam era Veteran status
El-Serag et al. 2002^53^	Hepatitis C diagnosis and hospitalization between 1992–1999	Multiple psychiatric illnesses	• Case–control study • 22,341 HCV-infected patients from the Vietnam period with HCV compared to 43,267 controls without HCV • Several psychiatric, drug-, and alcohol-use disorders are commonly found among HCV-infected Veterans compared with those who are not infected
El-Serag et al. 2014^54^	Active hepatitis C viremia	Cirrhosis and hepatocellular carcinoma (HCC)	• Cohort study to determine the effect of race on the risk for cirrhosis and HCC • 149,407 patients (mostly Vietnam War Veterans) with active HCV viremia were examined • Hispanics with HCV are at a significantly higher risk, whereas African Americans are at a considerably lower risk of developing cirrhosis and HCC than are Non-Hispanic Whites

### Conditions Associated with Vietnam War Exposures That May Be Encountered in the Inpatient Setting and Related Considerations for Care

#### PTSD

We identified 29 articles relevant to hospitalists on PTSD (Table 4 covers PTSD as an outcome resulting from ≥ 1 exposures; Table 5 examines PTSD as an exposure/comorbidity associated with other conditions).

**Table 4:** Articles Examining PTSD as an Outcome

Author/year	Article summary
Bourn et al. 2016^55^	• Case–control study • 226 Vietnam-era Veterans versus 132 Operation Iraqi Freedom/Operation Enduring Freedom (OIF/OEF) Veterans with somatic symptoms and PTSD • OIF/OEF Veterans show more somatic symptoms associated with PTSD than Vietnam Veterans
Currier and Holland 2012^56^	• Retrospective study • Combat Veterans from the National Vietnam Veterans Readjustment Study with analyses based on available information from 1637 Veterans who had served in the Vietnam theater at any point during 1962–1975 • Combat loss (i.e., deaths of fellow servicemembers), independent of other stressors, is associated with functional impairments in Veterans after controlling for other factors
Dohrenwend et al. 2008^57^	• Retrospective study of the roles of exposures and vulnerability factors in war-related PTSD • 248 male Vietnam Veterans of different racial and ethnic backgrounds (94 majority white, 70 Black, and 84 Hispanic) • Black Vietnam Veterans had higher rates of PTSD that were likely associated with greater severity of exposures experienced during wartime, prejudice, and negative homecoming. Hispanic Veterans also had higher rates of current PTSD than White Veterans, but differences were explained by these Veterans’ younger age and lower levels of education prior to their service
Dohrenwend et al. 2013^33^	• Retrospective study that examined the relationships of combat exposure, pre-service vulnerability, and involvement in harming civilians or prisoners with PTSD • 260 men from the National Vietnam Veterans Readjustment Study • Severity of combat exposure was associated with development of PTSD, while higher pre-war Vulnerability increased its persistence
Doron-LaMarca et al. 2015^38^	• Prospective study that examined fluctuations in PTSD symptoms over time • Sample of 34 male Vietnam-era Veterans who served in the Vietnam theater • Hyperarousal predicts fluctuations in other PTSD symptom clusters (reexperiencing, avoidance, and emotional numbing)
Goldberg et al. 2016^47^	• Cross-sectional study survey • 5598 male Vietnam era Veteran twins were assessed for PTSD, with comparisons by service with the Vietnam theater with those who did not serve in theater and by age at the time of interview (< 60 years versus ≥ 60 years) • Vietnam theater Veterans ≥ 60 years had a lifetime PTSD prevalence of 16.9% versus 5.5% among those who did not serve in the theater. For those younger than 60 years, prevalences were 22.0% and 15.7%, respectively
Koenen et al. 2003^58^	• Prospective survey study • 1377 American Legionnaires who had served in Southeast Asia during the Vietnam War and were followed over a 14-year period • Combat exposure most strongly predicted incidence and chronicity of PTSD, but perceived negative attitudes of others when returning home, minority race, and depression also predicted a more chronic course of PTSD
Kumar et al. 2022^59^	• Retrospective data analysis • 149 US military combat Veterans who served in the Vietnam War or Operation Enduring Freedom/Operation Iraqi Freedom/Operation New Dawn (OEF/OIF/OND) • Initial post-traumatic stress symptoms are linked to suicidal ideation, but social connectedness and engagement mitigate this risk, even years later
Magruder et al. 2015^60^	• Prospective survey study • 8742 women who were active-duty military personnel in the US Armed Forces at any time from July 4, 1965, through March 28, 1973, to study onset and prevalence of PTSD over the lifetime among those serving in Vietnam, near Vietnam, or in the United States only • Vietnam service increased PTSD risk for women Veterans due to wartime stressors, including sexual harassment
Magruder et al. 2016^61^	• Prospective study examining the long-term trajectory of PTSD • 4138 Veterans from the VET Registry, a national sample of male twin pairs from all service branches who served on active duty during the Vietnam era • Current PTSD prevalence is higher among Vietnam theater Veterans than non-theater Veterans for both late onset PTSD (6.55% theater, 3.29% non-theater) or chronic PTSD (3.95% theater, 1.16% non-theater)
Marmar et al. 2015^48^	• Prospective study • 1450 Vietnam Veterans with combat theater exposure to assess prevalence, course, and comorbidities of warzone PTSD across a 25-year period • Up to 271,000 Vietnam Veterans may still suffer from full or subthreshold PTSD, and many also experienced major depression four decades after the end of the war
Mohamed et al. 2021^62^	• Case–control study • 160 Vietnam war Veterans comparing neuropsychological measurements of white matter abnormalities among cohorts of those diagnosed with moderate to severe TBI, with PTSD, with both TBI and PTSD, and controls with neither diagnosis • PTSD and TBI Veterans show cognitive deficits and brain abnormalities decades after their traumatic experiences
Ney et al. 2021^63^	• Cross-sectional study • 72,698 Vietnam Veterans to examine differences and similarities between those diagnosed with post-traumatic headache versus those diagnosed with TBI who later developed headache disorder • Vietnam Veterans were among the group more likely to be diagnosed with post-traumatic headache
Ortega and Rosenheck 2000^64^	• Retrospective secondary data analysis • 1195 Vietnam Veterans with PTSD including 176 Mexican American, 61 Puerto Rican, and 35 other Hispanic Veterans • Hispanic Vietnam Veterans, especially Puerto Ricans, reported more severe PTSD symptoms than non-Hispanic whites, possibly reflecting differences in expressive style rather than illness severity
Renshaw et al. 2014^65^	• Cross-sectional in-person interview study • 465 Vietnam war Veterans evaluating gender differences in the associations of PTSD symptom clusters with relationship distress among Vietnam Veterans and partners • Both female Vietnam Veterans and female partners of male Vietnam Veterans reported greater distress associated with emotional numbing and/or withdrawal symptoms than their male counterparts
Schnurr et al. 2003^66^	• Cross-sectional study of the chronicity of PTSD among • 530 male and female Veterans who served in the Vietnam theater with PTSD • PTSD manifests in four clusters–remitted; chronic, late onset; chronic, intermittent; and chronic, unremitted. Women and minority men showed higher chronicity
Steenkamp et al. 2017^39^	• Cross-sectional study • 699 Vietnam war Veterans 40 years after the war using a self-report health questionnaire survey and a computer-assisted telephone health interview survey to assess predictors of development and severity of warzone-related PTSD • African-American race, lower education, poor homecoming reception, low social support, and recent stressors are consistent predictors of PTSD severity
Weiner et al. 2017^67^	• Cross-sectional study • 180 Vietnam Veterans with history of TBI and/or PTSD • Early results do not indicate an increased risk for Alzheimer’s in Veterans with TBI or PTSD based on amyloid PET imaging
Yoder et al. 2012^68^	• Cross-sectional study • 112 Veterans from different service eras, including 34 Vietnam-era Veterans, who experienced prolonged exposure therapy for combat-related PTSD • Vietnam Veterans responded well to exposure therapy

**Table 5:** Articles Examining PTSD as an Exposure Associated with Other Conditions

Author/year	Condition	Article summary
Boscarino 2008^69^	Heart disease (HD), all-cause mortality	• Prospective study • Vietnam Veterans who served in the Vietnam theater (2490) and those who did not serve in theater (1972) using phone interviews • PTSD was prospectively associated with heart disease (HD) mortality among Veterans free of HD at baseline
Forsberg et al. 2022^70^	Suicidality	• Retrospective cohort study • 14,401 male Vietnam Veteran twins comparing those who served in the Vietnam theater with those who did not • Vietnam Veterans had a similar suicide risk as non-theater Veterans, with no direct combat-suicide link. However, increased PTSD symptom severity was associated with a higher suicide risk
Goldberg et al. 2014^71^	Diminished health functioning and increased disability	• Cross-sectional study • 5574 male Vietnam-era Veteran twins • Both PTSD and combat exposure were independently associated with diminished mental health functioning and disability. Vietnam-era Veterans with PTSD have diminished functioning and increased disability
Johnson et al. 2004^72^	Violence, substance abuse, all-cause mortality	• Prospective study • 47 treatment-seeking male Veterans with combat-related PTSD • Veterans showed improvements in coping with chronic illness, reducing violence and substance abuse, but high PTSD symptom levels persist. The high mortality rate (2.8% per year) emphasizes the seriousness of PTSD, and inpatient psychiatric treatment programs show benefit
Kagan et al. 1999^73^	Coronary artery disease risk factors	• Case–control study • 73 Vietnam War Veterans suffering from PTSD who were consecutively admitted to a 90-day specialized inpatient PTSD unit • Elevated cholesterol, low-density lipoprotein, triglycerides, and reduced high-density lipoprotein were frequent in Vietnam Veterans with chronic PTSD
Kelley-Cook 2016^74^	Use of benzodiazepines, antipsychotic medications, hypnotics	• Retrospective pilot study • 176 US Vietnam Veterans at the end of live, 39 of whom were diagnosed with PTSD and 37 controls • Vietnam Veterans with PTSD at the end of life tend to receive more antidepressants and hypnotics. They often had more comorbid anxiety, depression, and insomnia
McFall et al. 1999^40^	Violent behavior (property destruction, threats with or without weapons, physical fighting)	• Prospective case–control study • 228 male Vietnam Veterans seeking inpatient treatment for PTSD • Vietnam Veterans with PTSD seeking inpatient treatment displayed higher levels of violent behavior compared to other inpatients
Mohamed et al. 2019^75^	Tauopathy	• Cross-sectional study • 80 Vietnam war Veterans with history of war-related TBI and/or PTSD comparing cognitive function and MRI/PET results between 4 groups: those with TBI, those with PTSD, those with both, and controls • A history of TBI and/or PTSD is associated with increased tauopathy resembling AD-typical and atypical patterns, and is correlated with impaired neuropsychological function
Schlenger et al. 2015^51^	Mortality	• Prospective mortality assessment • 2348 Vietnam era Veterans using National Vietnam Veterans Readjustment Study data to identify potential risk factors for mortality associated with Vietnam era military service • High exposure to warzone stress independently increased mortality risk for both male and female Vietnam theater Veterans, especially for those with a high likelihood of PTSD

Approximately 271,000 Vietnam Veterans have full or subthreshold PTSD,^48^ highlighting the need for continued outreach.^47,61^ To better connect with Vietnam Veterans, hospitalists should be aware most had very different experiences than civilians.^76^

Vietnam Veterans’ PTSD symptoms—hyperarousal, reexperiencing, avoidance, and emotional numbing^38^—are similar to Veterans of other military campaigns.^55,68^ PTSD significantly affects women Vietnam Veterans,^60^ though presentations are often delayed.^66^ Interestingly, Hispanic Vietnam Veterans have a higher risk for PTSD and experience more severe PTSD than non-Hispanic Vietnam Veterans.^64^ PTSD was prospectively associated with heart disease^69^ and dyslipidemia,^73^ and end-of-life Veterans often had more anxiety, depression and insomnia.^74^Additionally, traumatic brain injury (TBI) has potential associations with PTSD. The risk of Alzheimer’s dementia in patients with TBIs and PTSD is still being determined^62,67,75^ and TBI headaches could be a less recognized form of PTSD.^63^

Predisposing factors for development of PTSD included African-American race, lower education level, perceived poor homecoming reception and poor current social support, and experiencing more past-year stressors.^39^ Harming civilians or prisoners,^33^ severity of combat exposure,^33,71^ and bereavement^56^ were also predisposing factors.

Despite treatment, PTSD symptomatology among Vietnam Veterans was high and often required inpatient psychiatric treatment,^72^ neurotherapy,^77^ and interventions for anger management and aggression.^40^ Perceived social support,^58^ social engagement,^59^ and couples-based interventions^65^ positively influenced PTSD recovery.

#### Cancer

Seventeen of 19 papers discussing cancer focused on associations with Agent Orange/herbicide exposure (Table 2). The remaining two studies found no association of Vietnam service with HCV genotypes^78^ or gynecologic cancers.^79^

#### Neurological and Psychiatric

Thirty-five papers addressed neuropsychiatric disorders in Vietnam Veterans (Table 6; see Tables 4 and 5 for articles specific to PTSD).

**Table 6:** Articles Examining Neurological and Psychiatric Conditions

Author(s)/year	Condition/outcome	Article summary
Boscarino et al. 2018^80^	Mental health	• Chart review • 1730 community-based military Veterans in northeastern Pennsylvania was selected from a database and completed telephone interviews • Lower homecoming support may have an adverse effect on mental health decades after deployment. The study also found that low homecoming support was most prevalent among Vietnam Veterans. Acknowledging Vietnam Veterans service can build rapport
Brioschi Guevara et al. 2015^81^	Cognitive decline/TBI	• Observational study: on the basis of caregivers’ attachment style (secure, fearful, preoccupied, dismissing), participants with TBI were grouped into a high or low group. To examine the association between cognitive trajectory of participants with TBI and caregivers’ attachment style, they ran four 2 × 2 analysis of covariance on cognitive performances • 40 participants from the Vietnam Head Injury Study who sustained TBI • After controlling for other factors, cognitive decline was more pronounced in participants with TBI with a high fearful caregiver than among those with a low fearful caregiver. Additionally, it is important to provide caregivers support for TBI patients
Cypel et al. 2023^82^	Substance use (SU)	• Cross-sectional retrospective case–control study • 18,866 Veterans and 4530 non-Veterans to compare the prevalence of self-reported lifetime and current SU • SU is higher among Vietnam era Veterans versus non-Veterans. It is important to screen Vietnam Veterans for substance use disorders and to provide treatment
Eisen et al. 1998^83^	Physical health problems	• Retrospective, cross-sectional study • 4042 Vietnam era Twin Registry members were surveyed in 1987—combat exposure and physical health problems were self-reported by the Veterans • Combat experiences do not play a significant role in physical health problems in Vietnam Veterans
Ellsworth et al. 2021^84^	Terminal delirium	• Retrospective case–control study • 307 Veterans who expired while admitted for hospice care • Veterans at higher risk of receiving an antipsychotic for terminal delirium had the following conditions: liver disease; history of alcohol and/or drug abuse and liver disease; cancer and a mental health diagnosis; cancer and liver disease
Gould et al. 2015^85^	Depression and anxiety symptoms	• Cross-sectional study, phone or in person interviews • 6577 Veterans 50 years old and older from the 2006 Health and Retirement Study • In the community setting, the prevalence of elevated depression and anxiety symptoms are comparable between Veterans and non-Veterans. However, Vietnam Veterans were twice as likely to have elevated depression *and* anxiety symptoms. It is important to screen for and initiate treatment of depression and anxiety for Vietnam Veterans
Huang et al. 2018^86^	Depression	• Longitudinal cross-lagged twin difference study • 146 participants who wore an ambulatory electrocardiogram monitor for 24 h at the initial evaluation and at a 7-year follow-up visit • Reduced heart rate variability at baseline was associated with increased depressive symptoms at follow-up. There was also association with depression and lower heart rate variability at follow-up, but this was thought to largely be explained by antidepressant use. This really isn’t important to inpatient medicine
Na et al. 2023^87^	Psychiatric illness	• Retrospective data analysis • 767 Vietnam Veterans who took part in the National Health and Resilience in Veterans Study • Findings provide war-era specific characterization of the psychiatric status of US combat Veterans, which may help inform era-specific assessment, monitoring, and treatment of psychiatric disorders in the combat Veteran population
Smith et al. 2020^42^	Later-life health and functioning	• Mail survey; telephone interview • 4219 women Veterans who were active duty during the Vietnam era • Wartime stress exposures were associated with worse later-life health. Current PTSD was linked with lower health functioning and greater disability. Current major depressive disorder and generalized anxiety disorder were also associated with lower mental health functioning and greater disability. Screening for and treating PTSD in the inpatient setting can address later-life disability and can be a bridge to setting up outpatient resources
Wisco et al. 2017^44^	Mental disorders and suicidal ideation	• Retrospective cohort study • 564 Veterans who completed the National Health and resilience in Veterans Study and had a history of combat exposure and exposure to events during military service that violated their moral beliefs • Transgressions by self were associated with current mental disorders and suicidal ideation. All Veterans should be screened for suicidal ideation

While treating acute medical issues, hospitalists must often manage concomitant psychiatric disorders. Prior military service increases prevalence of these comorbidities, including cognitive impairment and dementia, potentially resulting from TBI or combat exposure (Table 7).

**Table 7:** Synthesis of Considerations for Hospitalists

Condition	Considerations
Agent Orange/herbicide exposures	• Hospitalists should prioritize prostate cancer screening and its complications in Vietnam Veteran patients when planning transitions of care and ambulatory follow-up • For those with clinical presentations concerning for prostate cancer, incorporating Agent Orange exposure history into a physician’s clinical decision making may help better predict clinically significant prostate cancer. Physicians should consider increased bladder cancer surveillance and prompt treatment of bladder cancer for Veterans with Agent Orange exposure • Hospitalists should be vigilant for signs of cognitive decline in this population, exercise appropriate delirium precautions for such inpatients, and ensure targeted screening and/or specialist follow-up after discharge for early detection and management strategies • In undifferentiated patient presentations of these conditions, a history of Agent Orange exposure could increase a hospitalists’ suspicion for peripheral neuropathy and neurodegenerative diseases such as Alzheimer’s disease and Parkinson’s disease • A National Academies of Science, Engineering, and Medicine review^22^ lists conditions with sufficient evidence of association with Agent Orange exposure, such as soft tissue sarcoma, non-Hodgkin lymphoma, chronic lymphocytic leukemia, Hodgkin lymphoma, and chloracne, and highlights conditions with limited or suggestive evidence of association, including laryngeal, prostate, lung, and bladder cancers, multiple myeloma, and AL amyloidosis, among others • Hospitalists should note that environmental studies suggest appreciable dioxin exposure is more likely in Veterans with repeated, long-term contact, such as those in Operation Ranch Hand or the Army Chemical Corps, and less likely for ground troops. However, Veterans who served in Vietnam may have been unknowingly involving the storage of Agent Orange and potential ground troop and other personnel’s exposure via incidents involving stored herbicide
Infectious disease	• Reported sex by men with men was a significant risk factor for hepatitis C among Vietnam Era Veterans • Major psychiatric disorders including depression, PTSD, psychosis, bipolar disorder, anxiety, alcohol and drug use disorders were more frequent among HCV-infected Veterans than in noninfected controls
Violence/combat exposures and service in the Vietnam theater	• Potentially morally injurious events experienced during combat can increased risk of mental disorders and suicidality ◦ The poor functional status of aging combat-exposed Veterans is of particular concern • Routine assessment and specialized interventions are recommended for anger and aggression in this population
PTSD	• Vietnam Veterans had a similar suicide risk as non-theater Veterans, with no direct combat-suicide link. However, increased PTSD symptom severity was associated with a higher suicide risk • PTSD was prospectively associated with heart disease (HD) mortality among Veterans free of HD at baseline. Consider PTSD in Vietnam war Veterans as a high risk for coronary artery disease • Increased severity of symptoms among Veterans with PTSD at the end of life suggests that treatment for these symptoms is needed
Neuropsychiatric conditions	• Stressors experienced by women Veterans during Vietnam-era military service can impact later-life function and disability • Autonomic dysregulation may affect depression risk. The number of reported psychiatric and substance use disorder diagnoses differ based on war-era. A significant percentage of Vietnam-era Veterans in this study reported alcohol and nicotine use disorders ◦ Prevalence of lifetime opioid and sedative use drug and alcohol use disorders, cannabis use, current"other drug"use, and nicotine use are high among the Vietnam Veteran population ◦ Severe pain, depression, and post-traumatic epilepsy were more strongly associated with drug use in Veterans than non-Veterans • The aging of Vietnam Veterans suggests that more late-life psychiatric services may be needed to improve functioning in conjunction with providing efficacious psychiatric treatment • Identify Veterans at a higher risk of delirium and initiate or refer for non-pharmacological management
Cancer	• In addition to offering age-appropriate screening, hospitalists should maintain a higher index of suspicion and referral to VA hospitals for patients with Agent Orange-related cancers including bladder cancer, soft tissue sarcomas (other than osteosarcoma, chondrosarcoma, Kaposi’s sarcoma, or mesothelioma), multiple myeloma, non-Hodgkin’s lymphoma, and chronic b-cell leukemias
Cardiovascular conditions	• Diabetes Mellitus Type 2, Ischemic Heart Disease, Hypertension and Hypothyroidism are more prevalent in Vietnam war Veterans who had “boots on the ground” (i.e., who served in the Vietnam theater), and these conditions have been recognized as related to Agent Orange exposure ◦ Consider referral to the VA for follow-up and to be entered into any VA registries for these conditions
Sexual/reproductive function	• Vietnam Veterans may have lower serum testosterone levels as a result of herbicide exposure
Respiratory	• No strong link between respiratory conditions and herbicide exposure was identified based on the articles reviewed here
Immunity/inflammation	• Biological markers associated with multiple inflammatory disorders were present in chronic PTSD patients • Recognize that PTSD can contribute to heightened immune response and inflammation
Other conditions: Skin care and amputation, sleep disturbances	• Vietnam Veterans continue to have skin complications at sites of amputations, contributing to significant morbidity and prosthesis abandonment ◦ Regularly assess amputation sites for skin issues like sores, infections, or dermatitis. Educate patients on proper skincare, hygiene, and using protective products. Ensure proper prosthesis fitting and adjust as needed to prevent discomfort • Screen for sleep disturbances, especially PTSD-related nightmares, and provide appropriate treatment • Coordinate care with dermatologists, prosthetists, pain specialists, and mental health providers. Schedule regular follow-ups to monitor skin health, prosthesis use, and sleep quality
Overall mortality	• The highest mortality risk was linked to warzone stress and probable PTSD • Agent Orange exposure increased risk of mortality from respiratory illness and malignancies • Vietnam Veterans consuming > 14 alcoholic drinks/week were at increased mortality risk • Religious and social engagement were protective factors

Patients with TBI exhibit brain MRI changes associated with poor verbal memory^88^ and cognitive dysfunction.^62^ Although some investigations revealed lesion patterns on brain CT imaging resembling those seen in Alzheimer dementia,^75^ similar findings have not been observed on amyloid PET scan.^67^ Trauma and head injuries can increase risk of anxiety^89^ and post-traumatic epilepsy.^45^ Combat exposure has been found to increase risk of suicide,^41,44^ diminished mental health functioning, and disability.^71^

In addition to dementia, Vietnam Veterans have been found to have a higher prevalence than non-Veterans of depression, PTSD, and alcohol use disorders,^90^ with the highest rates among those who served in the Vietnam theater.^46,82^ Neuropsychiatric diagnoses in Veterans have been identified as predictors of incident coronary heart disease.^7^ A cohort study of 4219 female Vietnam Veterans linked wartime stress to worse later-life health, with mental health conditions exacerbating disability.^42^

#### Cardiovascular Conditions

Ten studies on cardiovascular conditions were identified, primarily comprising cohorts or prospective studies with substantial sample sizes or longitudinal designs. One study of 4328 male Vietnam Veterans associated PTSD with heart disease mortality among those without prior heart disease, suggesting a connection between military service, PTSD, and early-onset heart disease.^69^ Another 20-year prospective study of 2105 Air Force personnel from Operation Ranch Hand evaluated herbicide exposure’s effects on health outcomes and revealed that depression, anxiety, hostility, and trait anger were the most significant predictors of coronary heart disease, with a composite psychological risk factor score being the strongest predictor.^7^ A 27-year longitudinal study of 787 male twin Veterans examined the relationship between depression and cardiometabolic health, finding early to midlife depression symptoms were associated with a higher risk of later self-reported health issues.^91^ Another longitudinal study, with baseline assessments of 166 Vietnam Era Twin Registry participants, investigated temporal association between depression and heart rate variability (HRV).^86^ The bidirectional relationship between depression and autonomic dysregulation, as measured by HRV, suggested autonomic function more strongly influences depression risk than vice versa, with antidepressant use as a key factor.

#### Sexual/Reproductive Function

Ten studies on sexual and reproductive health of varied design were identified. A case–control study comparing pregnancy outcomes between 4140 female Vietnam Veterans and contemporary Veterans revealed a significant link between military service in Vietnam and birth defects but not other pregnancy outcomes.^92^ In a case–control study of 7924 Vietnam and 7364 non-Vietnam Veterans, Vietnam Veterans reported higher rates of birth defects overall in their children, but total rates of major, minor, and suspected defects were comparable between both groups.^93^ A longitudinal study of 971 Operation Ranch Hand Veterans versus 1266 non-exposed Veterans showed a negative association between serum TCDD concentration and testosterone levels,^14^ and another found an association between dioxin exposure and increased sperm methylation age.^23^

#### Respiratory

Four articles^10,11,15,94^ on respiratory conditions were identified; all explored associations with herbicide exposure and found no strong links, as noted above.

#### Immunity/Inflammation

A prospective cohort study of 4255 Vietnam-era Veterans investigated correlation between serum Ig levels and mortality risk. Elevated Ig levels, particularly IgG, were associated with increased mortality from infectious diseases and other causes, suggesting potential underlying subclinical disease.^95^

A study of 2490 Vietnam Veterans examined the link between chronic PTSD and autoimmune diseases (e.g., rheumatoid arthritis, psoriasis, insulin-dependent diabetes, thyroid disease).^96^ Chronic PTSD was associated with these conditions, and Veterans with comorbid PTSD had elevated T-cell counts, immunoglobulin-M levels, dehydroepiandrosterone levels, and hyperreactive immune responses. Biological markers associated with multiple inflammatory disorders were present in chronic PTSD patients.^96^

#### Other Conditions

Four studies on limb loss and amputation were identified. One study comparing Operation Iraqi Freedom/Operation Enduring Freedom Veterans to Vietnam Veterans found no difference in quality of life related to use of prosthetics,^97^ though Vietnam Veterans reported continued pain and mental health complications from combat several decades later.^98^ Vietnam Veterans continue to have skin complications at sites of amputations, contributing to significant morbidity and prosthesis abandonment.^99^ Most disabled Veterans (from the Vietnam as well as other service eras) use a combination of assistive technologies for mobility.^100^

One study on sleep disturbances found frequent nightmares were specific for PTSD associated with warzone trauma.^49^

#### Overall Mortality

Eight studies on overall mortality were identified. Highest mortality risk was linked to warzone stress and probable PTSD.^51,72^ Agent Orange exposure increased risk of mortality from respiratory diseases and malignancies,^11,50^ and Operation Ranch Hand Veterans with Agent Orange exposure had increased mortality from cardiovascular causes.^16^ Vietnam Veterans consuming > 14 drinks per week faced increased mortality risk.^101^ Trauma-related mortality rates were higher in women Vietnam Veterans than non-Vietnam women Veterans,^102^ while religious and social engagement were protective factors.^103^

## DISCUSSION

This scoping review underscores the critical need for non-VA healthcare providers to recognize and address unique service-related exposures and presumptive conditions affecting Vietnam War Veterans, particularly from the standpoint of hospital-based practice, though managing these issues often spans both the inpatient and outpatient settings. Our focus on the inpatient perspective stems from our experiences as hospitalists and the increasing likelihood that non-VA physicians will encounter Vietnam Veterans hospitalized for these complex conditions.

Long-term health consequences of toxic exposures, particularly herbicides and other environmental hazards, include increased risk of cancers, respiratory diseases, neuropsychiatric disorders, cardiovascular conditions, and immune system dysfunctions. Despite well-documented associations, literature gaps persist, particularly regarding experiences of women Veterans and other underrepresented groups.

One key finding is the documented link between Agent Orange exposure and certain cancers, such as prostate cancer and soft tissue sarcomas. While evidence remains inconclusive for other conditions, early detection and intervention by non-VA providers can significantly improve patient outcomes. Furthermore, the high prevalence of PTSD and associated mental health disorders highlights the need for multidisciplinary approaches that integrate both psychological and physical healthcare.

The PACT Act expanded VA healthcare eligibility, facilitating access to specialized care, disability compensation, and preventive screenings. However, many Veterans remain unaware of these benefits. Non-VA providers play an essential role in identifying military service history, educating Veterans on their eligibility, and facilitating referrals to the VA. Strengthening collaborations between VA and non-VA healthcare systems is crucial in ensuring seamless transitions of care, optimizing health outcomes, and enhancing Veteran-centered services.

This review highlights the need for research into long-term treatment approaches for Veterans with service-related conditions. It remains unclear whether these conditions should be managed differently than similar conditions in non-Veteran populations. Addressing this gap will help refine treatment strategies and improve health outcomes.

This review is subject to limitations. As a scoping review, it does not quantify the strength of associations found in individual studies. Focusing solely on Vietnam Veterans may have missed important exposures and potential confounders (e.g., tobacco use, still highly prevalent among our Veteran population), and outcomes that occurred or have been better studied since the Vietnam War era, such as military sexual trauma.^104,105^ Finally, while we sought to include conditions that we believe are most likely to be encountered by physicians outside the VA system, our inclusion and exclusion criteria and study selection process may have been subject to unintentional bias based on our professional perceptions.

In conclusion, this scoping review provides valuable insights into service-related exposures and conditions affecting Vietnam War Veterans, offering non-VA healthcare providers a comprehensive resource to enhance understanding and care of this population. Bridging knowledge gaps between VA and non-VA healthcare providers is essential to ensure Vietnam War Veterans receive appropriate recognition, care, and access to VA benefits. By fostering education, collaboration, and research, healthcare providers can enhance quality of care for this population, ultimately fulfilling the promise of comprehensive and Veteran-centered healthcare.

## Supplementary Information

Below is the link to the electronic supplementary material.

Supplementary file1 (DOCX 69 KB)

Supplementary file2 (XLSX 100 KB)

## Data Availability

This scoping review is based on a review of published literature. All data analyzed during this study are available in the article and its supplementary materials.
